# Defining the full tomato NB-LRR resistance gene repertoire using genomic and cDNA RenSeq

**DOI:** 10.1186/1471-2229-14-120

**Published:** 2014-05-05

**Authors:** Giuseppe Andolfo, Florian Jupe, Kamil Witek, Graham J Etherington, Maria R Ercolano, Jonathan D G Jones

**Affiliations:** 1 The Sainsbury Laboratory, Norwich Research Park, NR4 7UH Norwich, UK; 2 Department of Agriculture Sciences, University of Naples ‘Federico II’, Via Universita’ 100, 80055 Portici, Italy

**Keywords:** RenSeq, NB-LRR, cDNA, Gene model, Disease resistance, Paralogous, Plant breeding, *Solanum lycopersicum*, *Solanum pimpinellifolium*, *Arabidopsis thaliana*

## Abstract

**Background:**

The availability of draft crop plant genomes allows the prediction of the full complement of genes that encode NB-LRR resistance gene homologs, enabling a more targeted breeding for disease resistance. Recently, we developed the RenSeq method to reannotate the full NB-LRR gene complement in potato and to identify novel sequences that were not picked up by the automated gene prediction software. Here, we established RenSeq on the reference genome of tomato (*Solanum lycopersicum*) Heinz 1706, using 260 previously identified NB-LRR genes in an updated Solanaceae RenSeq bait library.

**Result:**

Using 250-bp MiSeq reads after RenSeq on genomic DNA of Heinz 1706, we identified 105 novel NB-LRR sequences. Reannotation included the splitting of gene models, combination of partial genes to a longer sequence and closing of assembly gaps. Within the draft *S. pimpinellifolium* LA1589 genome, RenSeq enabled the annotation of 355 NB-LRR genes. The majority of these are however fragmented, with 5′- and 3′-end located on the edges of separate contigs. Phylogenetic analyses show a high conservation of all NB-LRR classes between Heinz 1706, LA1589 and the potato clone DM, suggesting that all sub-families were already present in the last common ancestor. A phylogenetic comparison to the *Arabidopsis thaliana* NB-LRR complement verifies the high conservation of the more ancient CC_RPW8_-type NB-LRRs. Use of RenSeq on cDNA from uninfected and late blight-infected tomato leaves allows the avoidance of sequence analysis of non-expressed paralogues.

**Conclusion:**

RenSeq is a promising method to facilitate analysis of plant resistance gene complements. The reannotated tomato NB-LRR complements, phylogenetic relationships and chromosomal locations provided in this paper will provide breeders and scientists with a useful tool to identify novel disease resistance traits. cDNA RenSeq enables for the first time next-gen sequencing approaches targeted to this very low-expressed gene family without the need for normalization.

## Background

To control pathogens, plants activate defence mechanisms that can culminate in a hypersensitive response (HR) in infected and adjacent cells [1]. Defence activation requires pathogen detection, which can occur outside or inside the plant cell, by one of two known distinct recognition mechanisms [2-4]. The first line of detection resides at the cell surface and involves recognition of pathogen-associated molecular patterns (PAMPs) through cell surface transmembrane receptors. Adapted pathogens have evolved mechanisms to overcome PAMP-triggered immunity (PTI) by suppressing the immune signalling using “effector molecules” [4]. Plants in turn possess a second line of defence, which is represented by proteins that detect specific effector molecules or their effects on host cell components. This mechanism is called ‘effector-triggered immunity’ (ETI). These intracellular immune receptors, termed *R* (resistance) genes, encode proteins that resemble mammal NOD-like receptors and typically carry a nucleotide binding and leucine-rich repeat domains (NB-LRR).

Plant NB-LRR proteins (also called NLR, NBS-LRR or NB-ARC-LRR proteins) are typically categorized into the TIR or non-TIR class, based on the identity of the sequences that precede the NB domain, as well as motifs within this domain [5]. The TIR class of plant NB-LRR proteins (TNLs) contains a Toll, interleukin 1 receptor, R protein homology (TIR) protein-protein interaction domain at the amino terminus. The non-TIR class (CNLs) is less well defined, but some members of this class contain helical coiled-coil–like (CC) sequences in their amino-terminal domain [1]. This class was previously grouped into sub-classes based on sequence similarity with the canonical CNLs that contain an EDVID amino-acid motif, and the RPW8-like proteins whose N-termini resemble the coiled-coil structure of the Arabidopsis RPW8 protein [6].

Tomato is the second most important vegetable crop worldwide (faostat.org), and breeding for disease resistance is a major goal. Several NB-LRR type *R* genes have been cloned from tomato, potato and pepper, and are used in current breeding efforts. The first draft tomato genome assembly revealed the large size of the NB-LRR gene family, and thus the potential *R* gene repertoire [7]. A first tomato *R* gene annotation [7] was reported based on the existing automated gene and protein predictions of the Tomato Genome Consortium [8].

Recently, we were able to show that the automated gene and protein predictions for the potato reference sequence failed to reveal over 300 potential NB-LRR genes in potato, using the Resistance gene enrichment and sequencing (RenSeq) approach [9]. The RenSeq method utilizes annealing between custom biotinylated 120-mer RNA probes that are designed based on Solanaceous NB-LRR sequences, with fragmented genomic DNA sequences of the plant of interest that have been ligated to Illumina adapters. After the non-bound fraction is washed away, the captured library, comprising ~50% NB-LRR sequences, can be amplified and sequenced on any next-generation sequencing platform, which facilitates obtaining sufficient sequence depth over the many NB-LRR genes that exist in multigene families [9]. However, even when RenSeq data was used to map the resistance to specific loci, it is still challenging to define the sequence of each paralogue in a multigene family.

In this study, we adopted an improved version of the RenSeq approach [7,9,10] in combination with Illumina MiSeq 250 bp paired-end sequencing on genomic DNA (gDNA) and on cDNA of the two sequenced tomato genomes *S. pimpinellifolium* LA1589 and *S. lycopersicum* Heinz 1706. RenSeq on gDNA allowed us to correct about 25% of the previously described tomato NB-LRR genes and to identify 105 novel genes from previously unannotated regions. We further report the first comprehensive study of the phylogenetic relationship between the individual NB-LRR genes in *S. pimpinellifolium* LA1589, *S. lycopersicum* Heinz 1706 and the Brassicaceae *Arabidopsis thaliana*. An important result for future applications of RenSeq was the reduction of sequence data complexity by enriching NB-LRR genes from cDNA, thus avoiding sequence analysis of non-expressed paralogues.

## Results and discussion

### Design and application of a tomato and potato RenSeq bait-library

In an effort to reannotate the NB-LRR gene complements of the sequenced tomato genomes *Solanum lycopersicum* Heinz 1706 and *S. pimpinellifolium* LA1589 (hence referred to as Heinz 1706 and LA1589, respectively), we designed an updated version of our customized RenSeq bait-library for NB-LRR gene targeted sequence enrichment [9]. This version of the bait-library comprises 28,787 unique 120-mer baits designed from the 260 and 438 NB-LRR-like sequences that were previously described from the tomato and potato genomes (prior Jupe et al. (2013), [9]), respectively (Additional file 1) [7,10]. The RenSeq experiment was carried out on genomic DNA, to facilitate the reannotation of the full NB-LRR complement, and in addition on double-stranded cDNA, to test if the complexity of sequencing data for this multigene family can be further reduced by only sequencing the expressed genes. Up to five barcoded samples were combined in one SureSelect NB-LRR capture reaction, and further pooled to up to 12 single samples prior sequencing.

The resulting RenSeq libraries with an average insert size of 700 bp were sequenced on a MiSeq platform (250-bp reads). For Heinz 1706, 9,395,874 reads were produced from gDNA. Of these, 50% (4,867,603) could be mapped to the 12 (plus ch00) reference tomato chromosomes, respectively (Table 1). Similarly, for LA1589, 4,980,032 reads were derived from the MiSeq run and 34% (1,680,734) mapped to the superscaffolds. Analysis of un-mapped gDNA derived reads revealed some sequence contamination from mitochondrial and chloroplast DNA, as reported earlier [9].

**Table 1:** Identification of novel NB-LRR genes from RenSeq data

	**Mapping**	**Andolfo et al.**[7]	**Novel**	**Total**
	**Heinz 1706 reads**	**Annotation**	**NB-LRR**	**NB-LRR**
**Ch00**	823,314	3 (2)	1	3
**Ch01**	369,154	17 (14)	7	21
**Ch02**	383,004	24 (16)	7	23
**Ch03**	334,034	8 (6)	3	9
**Ch04**	430,876	55 (40)	16	56
**Ch05**	495,739	39 (34)	11	45
**Ch06**	361,718	19 (17)	3	20
**Ch07**	202,113	21 (11)	8	19
**Ch08**	276,354	13 (11)	2	13
**Ch09**	230,882	16 (14)	9	23
**Ch10**	247,821	23 (19)	8	27
**Ch11**	451,661	34 (22)	20	42
**Ch12**	260,933	22 (15)	10	25
**Total**	**4,867,603**	**294 (221)**	**105**	**326**

BWA mapping of NB-LRR-enriched Illumina PE 250-bp MiSeq-reads to the reference *S. lycopersicum* Heinz 1706 aided the verification of previously reported NB-LRR genes [7] (verified genes in brackets), as well as the identification of novel NB-LRR encoding sequences.

### RenSeq data enables NB-LRR gene reannotation in Heinz 1706 and LA1589

To locate all potential NB-LRR encoding regions, gDNA RenSeq reads were mapped to the corresponding reference genome. Sequences with read coverage higher than 20× over a minimum of 45 nucleotides were identified, and resulted in a total of 7,290 and 6,465 genomic fragments from Heinz 1706 and LA1589, respectively, that were extracted with a 500 bp extension to both ends. Overlapping sequences were concatenated and used in a MAST search to identify amino acid motif compositions that are similar to NB-LRR genes [9,10]. This resulted in a total of 326 and 355 potential NB-LRR sequences from Heinz 1706 and LA1589, respectively (Table 2, Additional files 2, 3 and 4). All identified sequences were submitted to the *Plant Resistance Gene Wiki* (http://prgdb.crg.eu/wiki/Main_Page), from where they can be downloaded or used in BLAST searches.

**Table 2:** **Numbers of *S. pimpinellifolium *** **LA1589 and *S. lycopersicum *** **Heinz 1706 genes that encode domains similar to plant R proteins as identified in this study**

	**Protein domains**	***S. pimpinellifolium *** **LA1589**	***S. lycopersicum *** **Heinz 1706**
Full-length	CC-NB-LRR	110	195
	TIR-NB-LRR	14	26
**Total full-length**		124	221
Partial	CC-NB	33	14
	TIR-LRR	1	1
	TIR-NB	7	3
	NB	122	57
	TIR	12	10
	LRR	56	20
**Total partial**		231 (124*)	102
**Total**		355	326

*Partial *S. pimpinellifolium* LA1589 NB-LRR genes were considered fragmented, and thus part of a full not yet combined gene, when they are located within 500 bp of the beginning or end of a contig.

Using the available MAST motifs, genes could be classified as TNL or CNL, and presence/absence of motifs allowed conclusions to whether the identified gene is partial or full-length. In comparison to previous efforts [7,11], the RenSeq approach established 105 and 126 additional NB-LRRs within the Heinz 1706 and the LA1589 genome. About 70% (221) of all Heinz 1706 NB-LRR genes are potentially full-length, while in *S. pimpinellifolium* LA1589 only 37% (124) of the total NB-LRR complement (Tables 1 and 2) encodes the minimal domain structure (NB-ARC and LRR) necessary for a full-length gene. This is unlikely to reflect the true structure and might be due to the fragmented nature of the LA1589 genome, since about 35% (124) of the partial genes are fragments found at the border of contigs, whose missing counterparts are anticipated to lie on other contigs. Positional information of the motifs that are either associated with an N-terminal domain or the beginning of the NB-ARC were further used to predict the putative start codon, and the last LRR specific motif and reading frame information to establish the stop codon for potentially full-length sequences (Table 2 and Additional file 2).

### Correction of NB-LRR gene models in Heinz 1706

**Figure 1:**
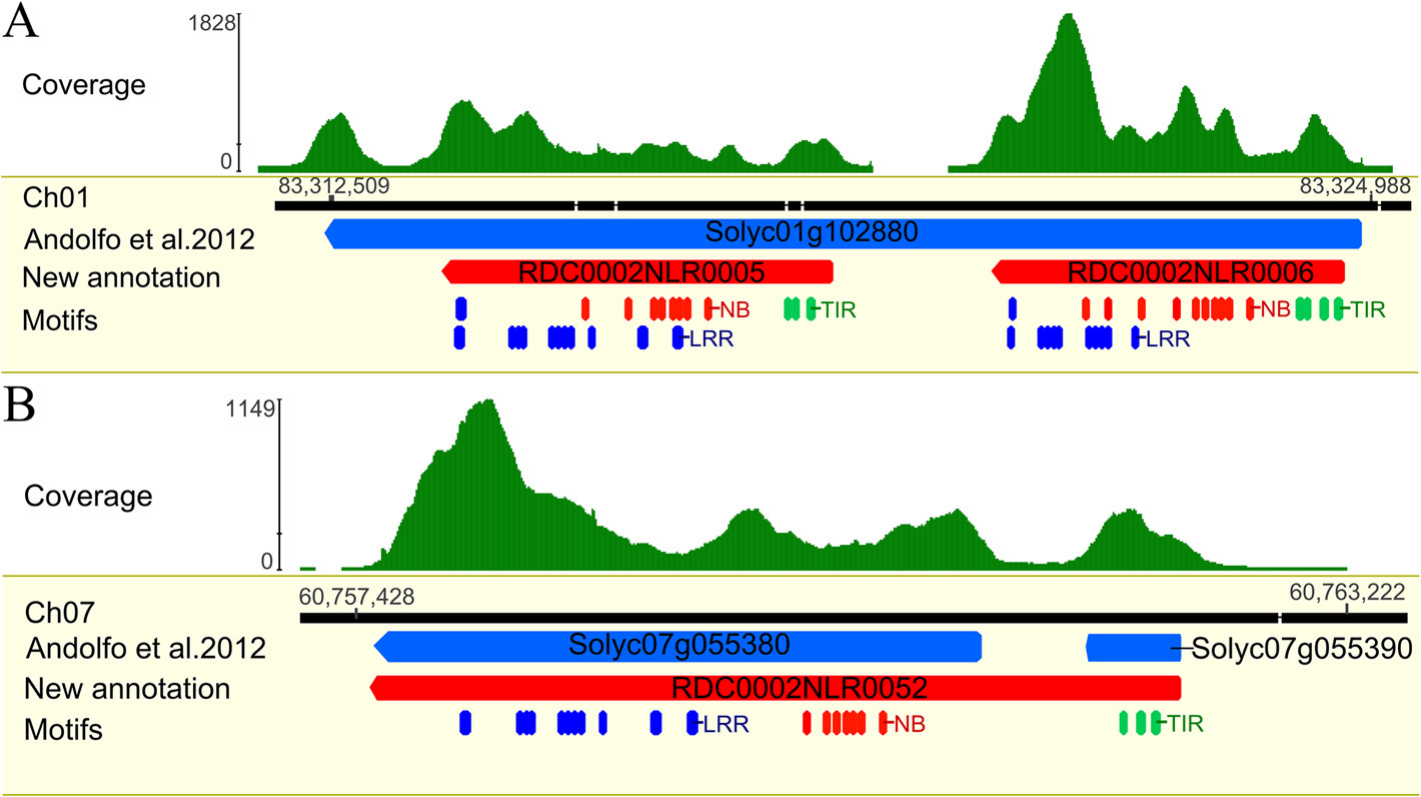
**Reannotation of two erroneously fused/split NB-LRR genes. (A)** Mapping of RenSeq reads identified two distinct patterns within Solyc01g102880, suggesting a fusion of two genes (blue box); **(B)** In contrast, Solyc07g055380 and Solyc07g055390 are predicted individual genes (red box), however a gap-free RenSeq read coverage pattern suggested that both are part of one longer sequence. The corrected annotation was confirmed in a MAST analysis using NB-LRR specific MEME motifs (TIR, NB and LRR motifs are shown in green, red and blue boxes, respectively [10]) and are depicted as boxed arrows (green) for the novel full-length TIR-NB-LRR genes RDC0002NLR0005, RDC0002NLR0006 and RDC0002NLR0052.

**Figure 2:**
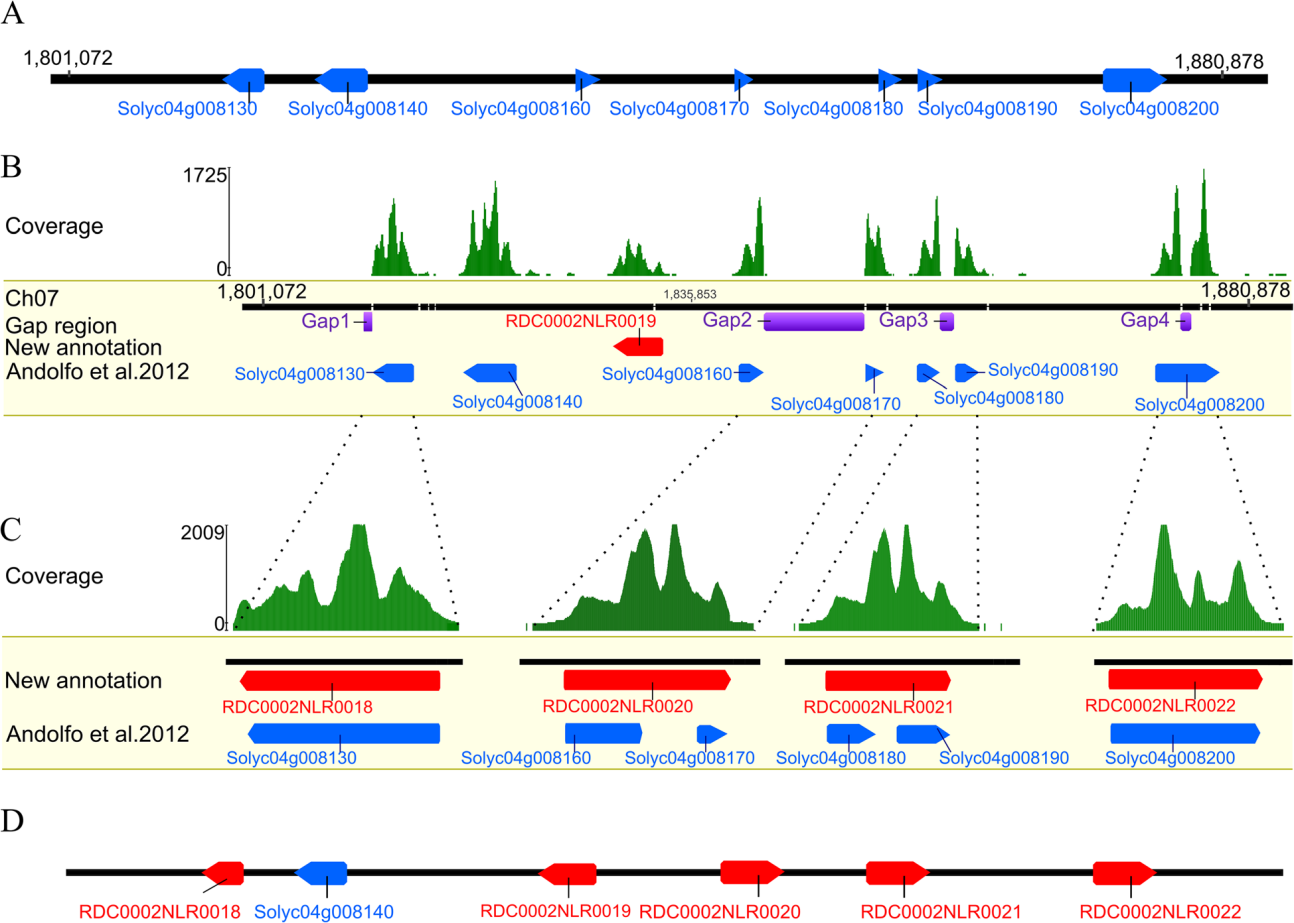
**Detailed analysis of a NB-LRR cluster between positions 1.81-1.87 Mb on chromosome 4. (A)** The Heinz 1706 region with annotations from Andolfo et al. [7]. NB-LRR genes are depicted as blue boxes. **(B)** Illumina MiSeq-platform RenSeq read coverage is shown with green peaks and identifies one yet unannotated NB-LRR RDC0002NLR0019 (red box). The purple boxes indicate stretches of N’s as unknown genomic sequences (Gap1 to Gap4, in violet). **(C)** Close-up of the analysed loci in which gaps were closed. Previous gene models (blue boxes), novel models (red boxes) and RenSeq read coverage (green peaks) are shown. **(D)** Representation of the reannotated NB-LRR gene cluster.

Our results identified 72 mis-annotated NB-LRR sequences compared to a previous study [7] in which an automated annotation was used (Table 1). Automated gene prediction software does not annotate all gene models correctly, and the efforts of genome sequencing consortia do generally not include the detailed verification of individual genes and gene families [7]. To fully reannotate the NB-LRR complement, we manually analysed all identified loci to correct erroneous start and stop codons, missing or additional exons, as well as erroneously fused or split genes (Additional file 5). In Figure 1A and 1B we present two examples of genes that were corrected using RenSeq data. Although the tomato genome is of high quality it still contains a number of regions with unknown sequence content, and among the annotated NB-LRR genes we found eight with stretches of N’s of varying length (between 97 and 7,851 bp). This number is significantly smaller than the 39 gaps found in potato NB-LRR sequences [9]. These gaps were filled by creating arches of sequence reads from both sides using the long 250 bp RenSeq reads, and the corresponding paired end information. An example is shown in Figure 2, where four sequence gaps were identified (Gap1–Gap 4, Figure 2B in violet) within a gene cluster on chromosome 4 that originally comprised three partial and four full-length NB-LRR genes [7]. Solyc04g008130 (CC-NB-LRR) had a gap at the expected stop codon position, which was then corrected. Two gaps were identified between the four partial NB-LRR genes Solyc04g008160, Solyc04g008170, Solyc04g008180 and Solyc04g008190, and closing of these enabled the reannotation of the partial genes into two full-size CC-NB-LRR genes (RDC0002NLR0020 and RCD0002NLR0021). Solyc04g008200 had a predicted gap of 784 nt in the middle of the sequence, that was corrected to 503 nucleotides. The RenSeq data further identified a novel NB-LRR in this cluster (RDC0002NLR0019, Figure 2B in red), and the final gene models are graphically depicted in Figure 2C. In comparison to Jupe et al. [9] who relied on 76 bp paired read data, the longer reads allowed a very rapid closure of the gaps with high confidence, using minimum numbers of reiterative mapping rounds.

### Conservation of the NB-LRR distribution between tomato and potato

**Figure 3:**
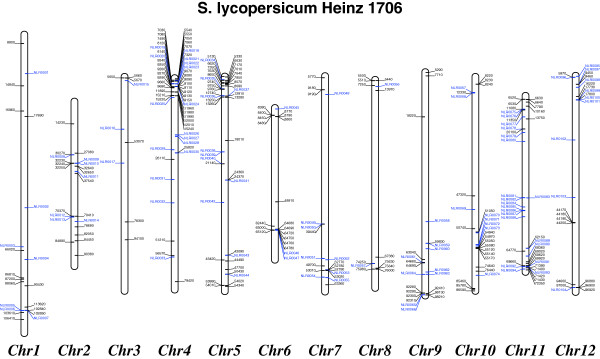
**Chromosomal distribution of Heinz 1706 NB-LRR genes.** The previously annotated NB-LRR genes [7] are shown in black and those discovered in this study are blue. Genes depicted to the left of the chromosome are on the forward strand and those on the right are on the reverse strand.

The genome-wide distribution of NB-LRR genes, based on the chromosome size, was significantly non-random (*χ*^2^ = 96, P <0.001) (Figure 3). The greatest numbers of NB-LRR genes are found on chromosomes 4, 5 and 11 (about 45% of the mapped genes), with the smallest number on chromosome 3 (9 genes), which is consistent with other Solanaceae including potato [9]. There was a clear difference between the genome distribution of the TNL and CNL genes, and the largest number of TNLs (43%) was found on chromosome 1, while TNLs are absent on chromosomes 3, 6 and 10. CNLs are however present on all chromosomes. The majority (about 66%) of the NB-LRR genes in tomato are organized in clusters (a region that contains four or more genes within 200 kb or less; [7]), including tandem arrays. We found 20 gene clusters that in total carry 107 NB-LRR genes, with on average five, and a maximum of 14 NB-LRR-encoding genes. The largest cluster was located on the short arm of chromosome 4 (Solyc04g009070 to Solyc04g009290) and resides in a ~110-kb-wide region.

It is intriguing that tomato has less than half of the number of NB-LRR genes compared to the doubled-monoploid reference potato. However, those present are found in syntenic chromosomal clusters between both species. Overall, the difference is not due to absence of gene sub-families, but due to a significantly smaller number of single genes within these clusters in tomato. Whole-genome duplication events did not contribute to the expansion in potato [8].

### Phylogenetic relationships between tomato NB-LRR genes

**Figure 4:**
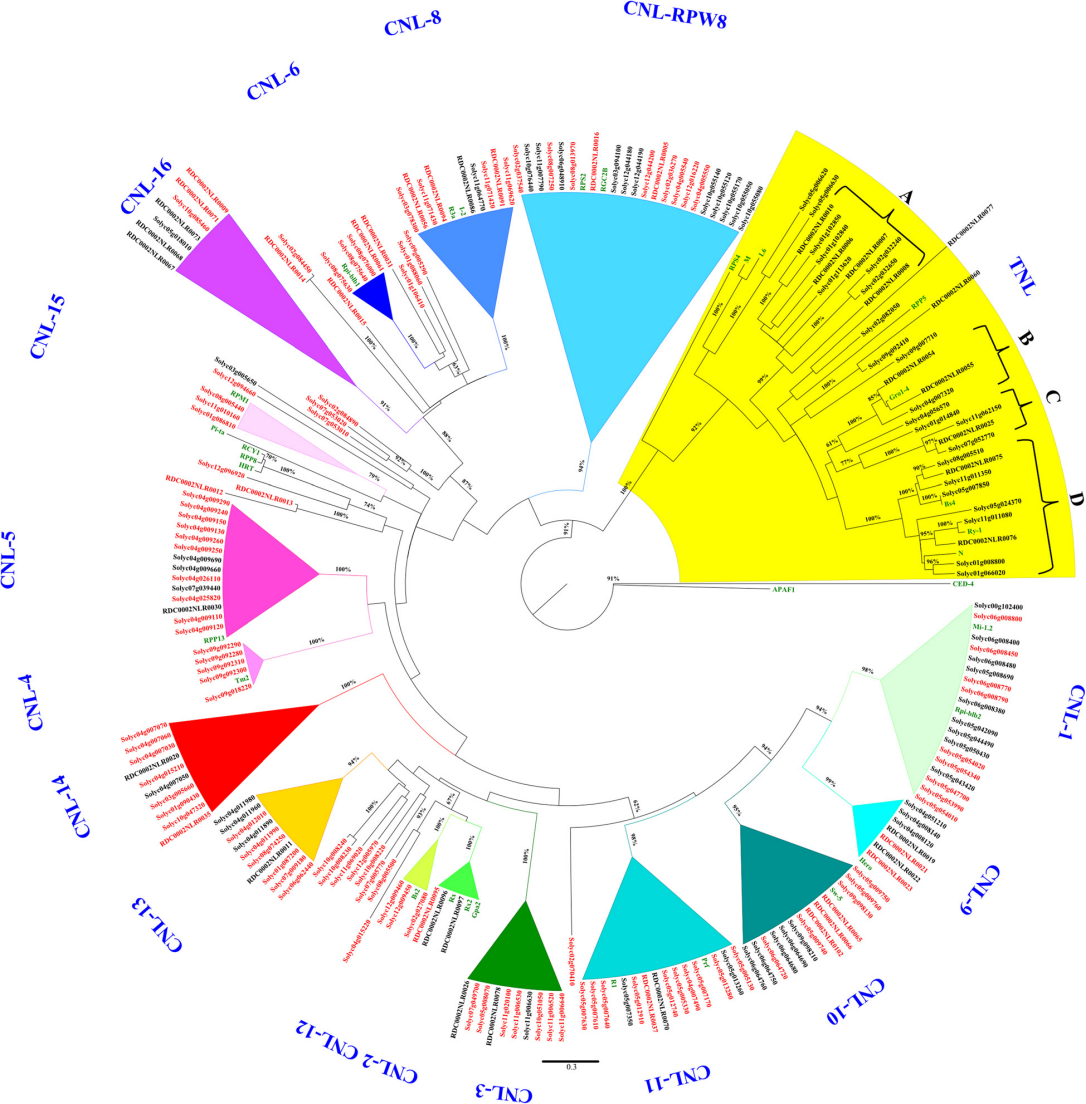
**Phylogenetic analysis of the reannotated Heinz 1706 NB-LRR genes.** Full NB-ARC domains of 240 reannotated NB-LRR genes were used together with 30 functionally characterized plant *R* genes (green font) to do a maximum likelihood analysis based on the Whelan and Goldman model. Clades are collapsed based on a bootstrap value over 79 and numerated. The TNL clade is drawn with a yellow background. Expressed genes, as identified by the cDNA RenSeq analysis, are in red font. Evolutionary analyses were conducted in MEGA5. Labels show the gene IDs (red for expressed NB-LRR genes; black for not-expressed genes). Bootstrap values higher than 79 (out of 100), are indicated above the branches. The tree is drawn to scale, with branch lengths proportional to the number of substitutions per site.

**Figure 5:**
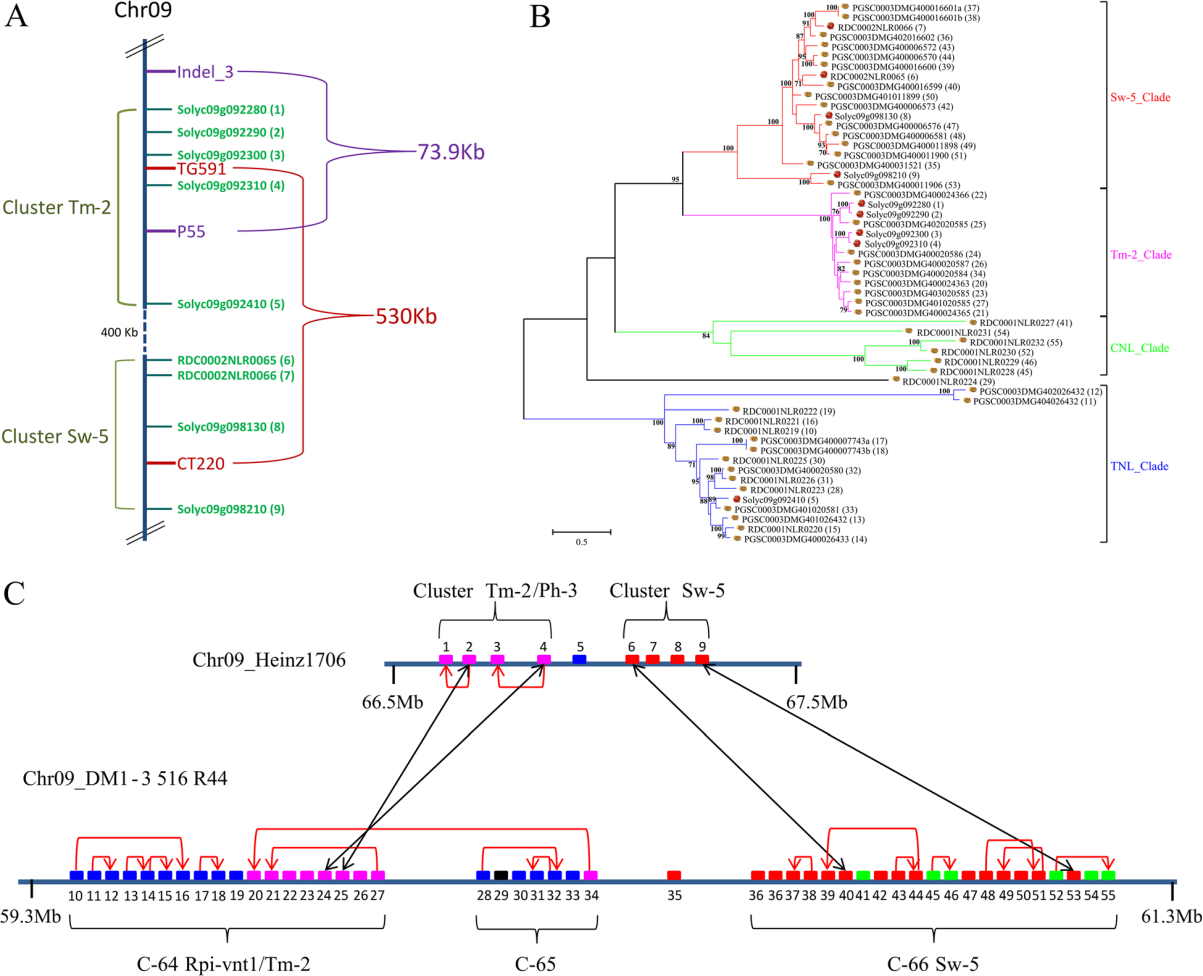
**Comparison of the *Tm-2 *and *Sw-5 *clusters between *Solanum lycopersicum *Heinz 1706 and *S. tuberosum *clone DM and identification of the *Ph-3 *locus in the tomato genome. (A)** Physical mapping position of NB-LRR gene clusters close to the physical *Ph-3* locus, based on marker information derived from [15,16]. **(B)** Phylogenetic analysis performed using the maximum likelihood method, based on the general time reversible model, for homologous sequences of the *Tm-2* and *Sw-5* clusters. Cartoon potatoes and tomatoes at the end of the branches indicate the origin of the sequence. Bootstrap values (100 replicates) are indicated above branches. **(C)** Schematic representation of hypothesised gene duplication events that occurred in the tomato and potato genomic region of *Tm-2* and *Sw-5* clusters. NB-LRR genes are depicted as boxes, and the colors relate to **(B)**.

The NB-ARC domain of NB-LRR genes has proven to be the most reliable protein domain with which to analyse phylogenetic relationships. Therefore the amino acid sequence of this domain was extracted from each NB-LRR gene with a full NB-ARC domain and used to perform a phylogenetic analysis for Heinz 1706 and LA1589 separately (Figure 4 and Additional file 6). For comparative purposes, we included 30 well-characterized cloned reference *R* genes from eleven different plant species and two out-group genes with a nucleotide-binding domain, the human *Apaf1.1* and nematode *Ced-4*, respectively (Additional file 7, green in Figures 4 and 5). A total of 240 and 222 NB-ARC domains of Heinz 1706 and LA1589 were aligned, respectively. The sequences were grouped into robust clades supported by bootstrap values ≥ 75%, and allowed the definition of 17 and 16 clades that have high sequence similarities in Heinz 1706 (Figure 4) and LA1589 (Additional file 6)*,* respectively.

The phylogenetic tree presents a clear distinction between TNL, CNL_RPW8_ and CNL_EDVID_ (CNL-1 to CNL-18) genes (Figure 4 and Additional file 6), as reported earlier for potato, and we also found this distinction to be very clear in Arabidopsis (Additional file 8) [5,6,10]. It is interesting to note that although this distinction is very conserved and points back to the last common ancestor, the included Solanaceae reference *R* genes share no similarity to any *A. thaliana* NB-LRR, and vice versa (Figure 4 and Additional file 8). Furthermore, Solanaceae CNL genes show a greater diversity and cluster expansion than TNL genes, which is in contrast to Arabidopsis and other Brassicaceae. Within the TNL group, three main subclades (A, B and D) were identified that are common between both analysed species. Members of subclade TNL-B and TNL-D share homology to functionally characterized *R* genes; the nematode resistance gene *Gro1.4* (*Solanum tuberosum*) and *Bs4*, *Ry1* and *N*, respectively. Subclade TNL-C with four members in Heinz 1706 is absent from LA1589.

Distinct from the canonical CNL_EDVID_ genes are those with a CC-domain similar to RPW8, that are suggested to have conserved functions and can be found throughout the plant kingdom [6]. The ancient position in the phylogenetic trees of tomato, potato and Arabidopsis, as well as other reports suggest that this group was present prior to the monocot/dicot split [6]. Well-characterized members of this clade are *N-required gene 1* (*NRG1*) from *N. benthamiana*, and the Arabidopsis *Activated Disease Resistance 1* (*ADR1*) gene.

Within the CNL_EDVID_ genes, 15 clades were defined in Heinz 1706 and 14 clades in LA1589 (Figure 4 and Additional file 6; clade IDs correspond between the two analysed species and potato [10]). Clade CNL-1 comprises *Mi1.2*, *Rpi-blb2* and similar sequences on chromosomes 5 and 6. It is interesting to note that clade CNL-1 shares a common ancestor with clades CNL-9 and CNL-10 (supported by 93% bootstrap indexes), which comprise members of the *Hero* family encoded on chromosome 4 and the *Sw-5* family on chromosome 9, respectively. Within the LA1589 phylogenetic tree these first three similar clades (CNL-1, CNL-17 and CNL-10) are less well defined, and *Hero* has only two similar sequences (RDC0003NLR0189 and RDC0003NLR0120) that were not considered a clade. Differences like these are likely due to the poor quality of the LA1589 genome assembly and the fragmented nature of genes annotated from this. CNL-11 shares in both phylogenetic trees similarities with *R1* and *Prf*, and all sequences are located on chromosome 5. Two small clades present in Heinz 1706 and LA1589 are CNL-2 and CNL-12 that share similarity to the characterized genes *Rx*, *Rx2* and *Gpa2*, and *Bs2*, respectively. Five individual large clades (CNL-3, CNL-13, CNL-14, CNL-16 and CNL-18) do not have similarity to any functional *R* gene, and might thus be potential sources of novel resistances. Clade CNL-4 includes the reference protein *Tm-2* and highly similar sequences encoded on chromosome 9 in both species. 14 and 10 genes similar to the *A. thaliana RPP13* were clustered in Heinz 1706 and LA1589*,* respectively, and can be found in clade CNL-5. Unique to tomato is CNL-15, which includes sequences similar to *RPM1*. CNL-16 harbours seven and eight genes from Heinz 1706 and LA1589, respectively. The small clade CNL-6 includes homologs of *Rpi-blb1* with high homology in both phylogenetic trees. Nine and 13 homologues of the very similar tomato *I2* and potato *R3a* genes are found in clade CNL-8 of Heinz 1706 and LA1589, respectively*.* Clade CNL-RPW8 is located on an ancestral position between TNL and CNL genes, and harbours the characterized genes *RPS2* and *RGC2B*[12,13].

### cDNA RenSeq significantly reduces the complexity of the NB-LRR gene complement

RenSeq was established as a tool to conduct targeted sequencing of the NB-LRR gene complement in order to identify polymorphisms that are linked to disease resistance between resistant and susceptible individuals of a segregating population [9]. For some NB-LRR sub-families, however, it is still challenging to define the many paralogous NB-LRR genes within chromosomal clusters and phylogenetic clades, and to identify the individual paralogue from which a co-segregating SNP derives. NB-LRR genes are not highly expressed, probably to prevent auto-immunity, and thus RNA-seq approaches would be unlikely to recover enough sequence depth. We tested whether the ability to enrich NB-LRR sequences 500-1000× using RenSeq could provide enough read depth to sequence cDNA of these low-expressed genes. A RenSeq experiment was carried out on double-stranded cDNA from mixed RNA samples of untreated and late blight (*Phytophthora infestans)*-infected Heinz 1706 and LA1589 leaves.

In total 2,882,986 paired-end 250-bp MiSeq reads were recovered from NB-LRR enriched Heinz 1706 cDNA; 65% (1,863,598 reads) of which map to the 12 reference chromosomes. Reads not mapping to the chromosomes, were identified to originate from ribosomal RNA. High-stringency Bowtie mapping, omitting reads that would map to more than one sequence (see Methods), placed 214,050 and 235,656 reads onto 167 Heinz 1706 and 154 LA1589 NB-LRR genes, respectively. On average 1281 and 1560 reads mapped per NB-LRR sequence. Several sequences had very low number of mapping reads (minimum of 2; Additional files 2 and 3) and might be mapping artefacts, but were still considered. Overall, the complexity of the NB-LRR complement was reduced by 51% in Heinz 1706 (Figure 4), and 43% in LA1589 (Additional file 6) and thus the number of paralogues of any candidate *R* gene that need to be analysed is halved. More importantly, this reduction was even over all phylogenetic clades. These data however do not allow any conclusions about a correlation between read number and expression level, as a certain bias from the bait-library cannot be excluded (though was not seen after RenSeq on gDNA). Of the expressed genes, 90% are full length and 10% are partial genes. The number of expressed partial genes is higher than seen for other plant species, and might suggest a role in NB-LRR gene regulation [14].

### Integrating genetics and genomics to locate best NB-LRR resistance gene candidates

Breeding for plant disease resistance is based on genetic mapping of resistance-conferring alleles. The results presented in this paper build a framework for an integration of genomics and genetics, by using available marker data in conjunction with positional and sequence information for the annotated NB-LRR genes. The following cases will present an example of a recently mapped but not yet cloned *R* gene, and another locus under high evolutionary pressure for which no *R* gene in tomato has been identified yet.

Two recent publications presented independently a set of four flanking markers for the *R* gene *Ph-3* that confers resistance to certain *P. infestans* isolates in *S. lycopersicum*[15,16]. Alignment based anchoring of these marker sequences (Indel_3, CT220, TG591 and P55) to the reference chromosomes identifies a 600-kb region on the short arm of Chromosome 9 (Figure 5A). This genomic region includes sequences with high similarity to the tomato *R* genes *Tm-2* and *Sw-5*, which confer resistance to Tomato mosaic virus (ToMV) and Tomato spotted wilt virus (TSWV), respectively. The *Tm-2* cluster in Heinz 1706 consists of four CC-NB-LRR genes that share over 90% pairwise identity. The *Sw-5* cluster is composed of three full length CC-NB-LRR and a partial CC-NB gene. Interestingly, the two independently identified marker pairs span a common region of only 30-kb, in which only one NB-LRR gene is located between TG591 and P55. The CNL Solyc09g092310 is the closest homologue in Heinz 1706 and is thus a potential candidate for *Ph3* in the resistant tomato line [15-17]. This CNL has an amino acid identity of 77.4% and 73% with Rpi-vnt1.1 and Tm-2, respectively. Figure 5C shows the syntenic conservation of the *R* gene clusters around the *Ph-3* candidate gene between tomato and potato [9]. A combined potato and tomato phylogenetic analysis of sequences found in this syntenic region did not result in a clear distinction of the sequences derived from both species, suggesting that these clusters were already present in the last common ancestor (Figure 5B). Four highly similar gene pairs with an identity between 82 and 89% (Figure 5C; black arrows) were identified that might be most ancestral.

**Figure 6:**
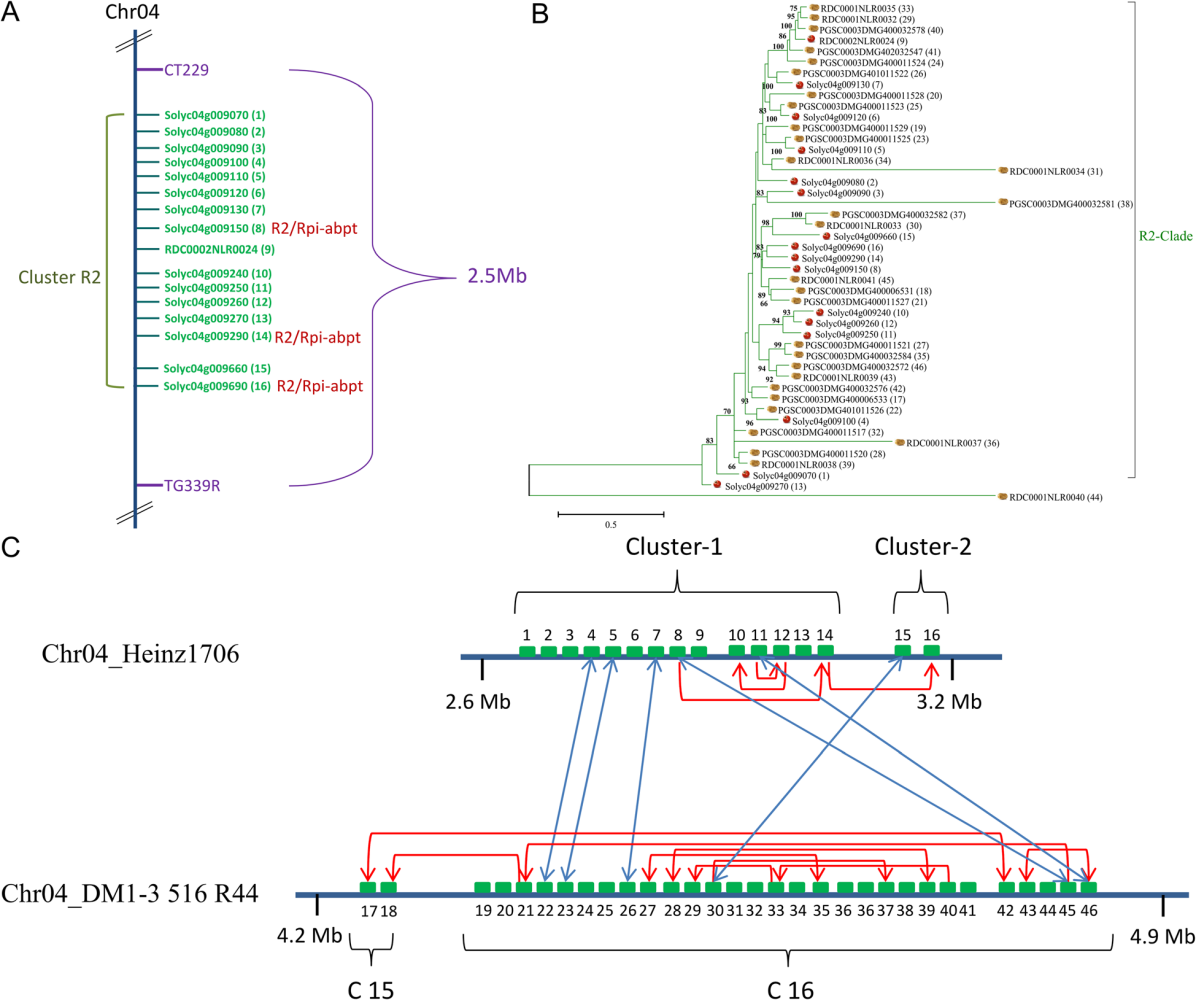
**The evolutionary history of the largest NB-LRR gene cluster involving 16 NB-LRR genes on chromosome 4. (A)** Physical mapping position of NB-LRR genes around the potato *R2* cluster. **(B)** The phylogenetic analysis was inferred using the maximum likelihood method based on the general time reversible model in MEGA5. Cartoon potatoes and tomatoes at the end of the branches indicate the origin of the sequence. Bootstrap values higher than 60 are indicated above branches. The tree is drawn to scale, with branch lengths measured in terms of the number of substitutions per site. **(C)** Schematization of the duplication events that occurred in these genomic regions. Arrows highlight the most probable gene duplication events. NB-LRR genes are depicted as boxes, and the colors relate to **(B)**.

Chromosome 4 of Heinz 1706 harbours the largest NB-LRR gene cluster with 14 members (all located in CNL-11) (Figure 6A). All members of this cluster share high sequence similarity to each other and the wild potato derived *R* genes *R2*, *Rpi-blb3* and *Rpi-abpt* that are located in a syntenic region of the potato chromosome 4 [18,19]. Synteny is also shown by mapping the markers CT229 and TG339R, both are linked to *Rpi-blb3*[17]. A detailed phylogenetic analysis of proteins encoded by members of these clusters from tomato and potato show that all genes fall into a unique clade with mean identities of 80% and a bootstrap value of 83% (Figure 6B). Solyc04g009290 has high sequence identity to *R2* (88%; Figure 6A). The phylogenetic tree further identifies nine duplication events in potato that must have occurred after the divergence of potato and tomato (Figure 6C). Microsyntenic analyses identified six NB-LRR genes with high sequence similarity between 78 and 85% in both species (blue arrows; Figure 6C)*.* No functional *R* gene has yet been identified in tomato from this rapidly evolving cluster, but it can be speculated that some alleles of this locus might encode valuable disease resistance.

## Conclusions

RenSeq facilitates deep sequencing and identification of the complete NB-LRR gene complement in plants. The Illumina MiSeq platform with 250-bp reads facilitates error-free closing of gaps in the assembly. We anticipate that carrying out RenSeq on other assembled plant genomes would increase the number of annotated NB-LRR sequences and will enable more targeted and specific resistance breeding strategies. While RenSeq on bulked resistant and bulked susceptible plants allows the identification of NB-LRR gene alleles that cosegregate with a resistance phenotype using “quick”-mapping or genotype-specific mapping, the list of candidate genes can further be reduced by cDNA RenSeq that limits the number of *R* gene candidates to be analysed to only those that are expressed. A combination of these methods will greatly accelerate the recruitment of natural resistance gene biodiversity for crop improvement.

## Methods

### Plant material and preparation of RenSeq libraries

Fully expanded leaves of *S. lycopersicum* Heinz 1706 and *S. pimpinellifolium* LA1589 were detached from 3-week old glasshouse grown plants. Three leaves were inoculated with two 20 μl-drops per leaflet of water, or a suspension of *P. infestans* isolate 2006_3928A (50,000 zoospores/ml). One inoculation spot per leaflet was harvested 24 hours post-inoculation as leaf discs with 10 mm in diameter, and frozen in liquid nitrogen. The remaining spots were observed at 6-dpi for successful colonisation with *P. infestans.* Leaf discs of both treatments were mixed and RNA was extracted using the TRI-reagent (Sigma-Aldrich) and Directzol RNA Mini-prep (Zymo Research), following manufacturers recommendations. First-strand cDNA was made using oligo-dT and random hexamer primers and First-Strand Superscript II (Sigma-Aldrich). The second strand was made as described in [20].

gDNA was extracted from young leave tissue of the same plants, using the DNeasy Plant Mini kit (Qiagen), following manufacturers recommendations.

Illumina MiSeq libraries were prepared using the NEBNext Ultra DNA library prep kit (NEB) using 2 to 3 μg starting material. Libraries were multiplexed using the NEBNext Multiplex Oligos for Illumina (Index Primers Set I). Up to three libraries were pooled and NB-LRR like sequences were captured as described in Jupe et al. [9] using a Agilent SureSelect kit with an updated bait library comprising 28,787 unique 120-mer oligos (Additional file 1). Enriched libraries were amplified up to 1 μg, and sent for MiSeq 250-bp paired end sequencing at The Genome Analysis Center (TGAC, Norwich Research Park, UK).

### Identification and annotation of NB-LRR genes in *Solanum spp*

All Illumina MiSeq data analysis was carried out using the Sainsbury Laboratory instance of the Galaxy project if not stated otherwise [21]. To identify and annotate NB-LRR loci in the Tomato genome [8], NB-LRR enriched paired-end Illumina MiSeq reads were mapped to the twelve chromosomes, using BWA version 0.5.9 (default parameters) (TGC_SL2.40_pseudomolecules.fasta). The mapping information (BAM-format) was imported into Geneious 6.0 and visualized per chromosome (http://www.geneious.com/). The Illumina read coverage over previously identified NB-LRRs was determined as described in Jupe et al. [9]. Potential full-length sequences were determined using the MAST output as described in Jupe et al. [10], and this was further used to identify start and stop positions for each gene. Gaps in the assembly were closed following the method described in Jupe et al. [9]. IDs for novel genes are as per definition in Jupe et al. [9] for the *R* gene discovery consortium (RDC) and include the species code RDC0002 (Heinz 1706) and RDC0003 (LA1589).

### Analysis of cDNA RenSeq libraries

Raw high-quality MiSeq reads were mapped to the reannotated NB-LRR gene complement using Bowtie version 0.12.7 under stringent conditions (reads mapping more than once are omitted). The resulting SAM-file was filtered for mapped reads and the number was counted per NB-LRR gene. No cut-off was applied to the number of mapping reads.

### Phylogenetic and gene duplication analysis

To identify the NB domain sequences used for the phylogenetic analysis, amino acid sequences of the NB domain of the reference *R* genes (reported in Additional file 7), were used to search in a BLASTx analysis with an expected value of <1e^−3^. Sequences with less than 50% of the full-length NB-ARC domain (Pfam database ID: PF00931) were excluded. Evolutionary analyses were conducted using MEGA5 [22]. The phylogenetic relationships of mapped NB-LRR genes were inferred separately (e.g., *S. lycopersicum* Heinz 1706 and *S. pimpinellifolium* LA1589 groups) using the maximum likelihood method based on the WAG model [23]. 162 *Arabidopsis thaliana* NB-LRR gene sequences were extracted from the TAIR database (http://www.arabidopsis.org/). For nucleotide sequences, the General Time Reversible Model was used. The bootstrap consensus tree inferred from 100 replicates was taken to represent the evolutionary history of the sequences analysed [24]. All the sequences were aligned using ClustalW 1.74 [25].

### Availability of supporting data

The data sets supporting the results of this article can be found as Additional files, and sequence reads are available in the European Nucleotide Archive, http://www.ebi.ac.uk/ena/data/view/ERP002644. All NB-LRR sequences can be found in Additional file 4 and were uploaded to the *Plant Resistance Gene Wiki* (http://prgdb.crg.eu/wiki/Main_Page), and can be accessed through searches for the RDC0002NLR or RDC0003NLR ID.

## Competing interests

The authors declare that they have no competing interests.

## Authors’ contributions

AG, FJ and KW designed the study. AG carried out the annotation and phylogenetic analyses. KW and FJ planned and carried out the RenSeq experiment. FJ analysed the cDNA RenSeq data. GJE contributed to the Bioinformatics analysis. AG and FJ wrote the manuscript. KW, ME and JJ critically reviewed the manuscript. All authors read and approved the final manuscript.

## Supplementary Material

Additional file 1 **Customized *R *** **gene enrichment and sequencing (RenSeq) bait library.** A total of 28,787 120-mer probes were designed on previously annotated NB-LRR genes of potato and tomato. Click here for file

Additional file 2 **Detailed information on *Solanum lycopersicum *** **Heinz 1706 NB-LRR.** This table contains information for 326 annotated Heinz 1706 NB-LRR genes, including strand information, whether the gene is expressed or not, start and end position on the chromosome and the protein class. Click here for file

Additional file 3 **Detailed information on *Solanum pimpinellifolium *** **LA1589 NB-LRR.** This table contains information for 355 annotated LA1589 NB-LRR genes, including strand information, whether the gene is expressed or not, start and end position on the contig/scaffold and the protein class. Click here for file

Additional file 4 ***Solanum lycopersicum *** **Heinz 1706 and *S. pimpinellifolium *** **annotated NB-LRR genes in FASTA format.** All identified members of the tomato NB-LRR gene family are included in this file. Click here for file

Additional file 5 **List of re-annotated Heinz 1706 NB-LRR genes.** This list details the original Heinz 1706 Solyc-IDs including the changes made after RenSeq analysis and the new gene IDs. Click here for file

Additional file 6 **Phylogenetic tree of *Solanum pimpinellifolium *** **LA1589 NB-ARC domains.** Evolutionary analyses were performed like in Heinz 1706, on the basis of the NB-ARC domain of 222 rennotated NB-LRRs. Labels show the gene IDs red for expressed NB-LRR genes; black for not-expressed genes. Click here for file

Additional file 7 **Reference NB-LRR genes for phylogenetic studies.** A list of 30 previously cloned and characterized plant NB-LRR genes and two outgroup genes that were used in the phylogenetic studies as reference genes. Click here for file

Additional file 8 **Phylogenetic tree of *Arabidopsis thaliana *** **NB-LRR genes.** Evolutionary analyses were performed like in *Solanum spp.* for 194 NB-ARC domains. Click here for file

## References

[B1] Heath MC Hypersensitive response-related death Plant Mol Biol 2000 44 321 334 10.1023/A:1026592509060 11199391

[B2] Dangl JL Jones JDG Plant pathogens and integrated defence responses to infection Nature 2001 411 826 833 10.1038/35081161 11459065

[B3] Chisholm ST Coaker G Day B Staskawicz BJ Host-microbe interactions: shaping the evolution of the plant immune response Cell 2006 124 803 814 10.1016/j.cell.2006.02.008 16497589

[B4] Jones JDG Dangl JL The plant immune system Nature 2006 444 323 329 10.1038/nature05286 17108957

[B5] Meyers BC Kaushik S Nandety RS Evolving disease resistance genes Curr Opin Plant Biol 2005 8 129 134 10.1016/j.pbi.2005.01.002 15752991

[B6] Collier SM Hamel LP Moffett P Cell death mediated by the N-terminal domains of a unique and highly conserved class of NB-LRR protein Mol Plant Microbe Interact 2011 24 918 931 10.1094/MPMI-03-11-0050 21501087

[B7] Andolfo G Sanseverino W Rombauts S Van der Peer J Bradeen JM Carputo D Frusciante L Ercolano MR Overview of tomato (Solanum lycopersicum) candidate pathogen recognition genes reveals important Solanum R locus dynamics New Phytol 2013 197 223 237 10.1111/j.1469-8137.2012.04380.x 23163550

[B8] The Tomato Genome Consortium The tomato genome sequence provides insights into fleshy fruit evolution Nature 2012 485 635 641 10.1038/nature11119 22660326 PMC3378239

[B9] Jupe F Witek K Verweij W Sliwka J Leighton P Etherington GJ Maclean D Cock PJ Leggett RM Bryan GJ Milne L Hein I Jones JDG Resistance gene enrichment sequencing (RenSeq) enables re-annotation of the NB-LRR gene family from sequenced plant genomes and rapid mapping of resistance loci in segregating populations Plant J 2013 76 530 540 10.1111/tpj.12307 23937694 PMC3935411

[B10] Jupe F Pritchard L Etherington GJ Mackenzie K Cock PJ Wright F Kumar Sharma S Bolser D Bryan GJ Jones JD Hein I Identification and localisation of the NB-LRR gene family within the potato genome BMC Genomics 2012 13 75 10.1186/1471-2164-13-75 22336098 PMC3297505

[B11] Xiaoxun N Jingjing Y Silong S Wencai Y Identification and analysis of resistance-like genes in the tomato genome J Phytopathol 2013 162 117 199

[B12] Bent AF Kunkel BN Dahlbeck D Brown KL Schmidt RL Giraudat J Leung JL Staskawicz BJ RPS2 of Arabidopsis thaliana: a leucine-rich repeat class of plant disease resistance genes Science 1994 265 1856 1860 10.1126/science.8091210 8091210

[B13] Shen KA Chin DB Arroyo-Garcia R Ochoa OE Lavelle DO Wroblewski T Meyers BC Michelmore RW Dm3 is one member of a large constitutively expressed family of nucleotide binding site-leucine-rich repeat encoding genes Mol Plant Microbe Interact 2002 15 251 261 10.1094/MPMI.2002.15.3.251 11952128

[B14] Marone D Russo MA Laido G De Leonardis AM Mastrangelo AM Plant nucleotide binding site-leucine rich repeat (NBS-LRR) genes: active guardians in host defense response Int J Mol Sci 2013 14 7302 7326 10.3390/ijms14047302 23549266 PMC3645687

[B15] Andolfo G Sanseverino W Aversano R Frusciante L Ercolano MR Genome-wide identification and analysis of candidate genes for disease resistance in tomato Mol breeding 2013 9928 7

[B16] Zhang C Liu L Zheng Z Sun Y Zhou L Yang Y Cheng F Zhang Z Wang X Huang S Xie B Du Y Bai Y Li J Fine mapping of the Ph‑3 gene conferring resistance to late blight (Phytophthora infestans) in tomato Theor Appl Genet 2013 126 2643 2653 10.1007/s00122-013-2162-1 23921955

[B17] Chunwongse J Chunwongse C Black L Hanson P Mapping of Ph-3 gene for late blight from L. pimpinellifolium L3708 Rep Tomato Genet Coop 1998 48 13 14

[B18] Li X Van Eck HJ van der Voort JNAM R Huigen DJ Stam P Jacobsen E Autotetraploids and genetic mapping using common AFLP markers: The R2 allele conferring resistance to Phytophthora infestans mapped on potato chromosome 4 Theor Appl Genet 1998 96 1121 1128 10.1007/s001220050847

[B19] Park T Gros J Sikkema A Vleeshouwers VGAA Muskens M Allefs S Jacobsen E Visser RGF van der Vossen EAG The late blight resistance locus Rpi-blb3 from Solanum bulbocastanum belongs to a major late blight R gene cluster on chromosome 4 of potato Mol Plant Microbe Interact 2005 18 722 729 10.1094/MPMI-18-0722 16042018

[B20] Okayama H Berg P High-efficiency cloning of full-length cDNA Mol Cell Biol 1982 2 161 170 6287227 10.1128/mcb.2.2.161-170.1982 PMC369769

[B21] Maclean D Kamoun S Big data in small places Nat Biotechnol 2012 30 33 34 10.1038/nbt.2079 22231089

[B22] Tamura K Peterson D Peterson N Stecher G Nei M Kumar S MEGA5: molecular evolutionary genetics analysis using maximum likelihood, evolutionary distance, and maximum parsimony methods Mol Biol Evol 2011 28 2731 2739 10.1093/molbev/msr121 21546353 PMC3203626

[B23] Whelan S Goldman N A general empirical model of protein evolution derived from multiple protein families using a maximum-likelihood approach Mol Biol Evol 2001 18 691 699 10.1093/oxfordjournals.molbev.a003851 11319253

[B24] Felsenstein J Confidence limits on phylogenies: an approach using the bootstrap Evolution 1985 39 783 791 10.2307/2408678 28561359

[B25] Thompson JD Higgins DG Gibson TJ Clustal w: improving the sensitivity of progressive multiple sequence alignment through sequence weighting, position-specific gap penalties and weight matrix choice Nucleic Acids Res 1994 22 4673 4680 10.1093/nar/22.22.4673 7984417 PMC308517

