# Supplementary figures and images for: Defining the full tomato NB-LRR resistance gene repertoire using genomic and cDNA RenSeq
Source: BMC Plant Biol. 2014 May 5;14:120. doi: 10.1186/1471-2229-14-120 (PMC4036795; doi:10.1186/1471-2229-14-120)

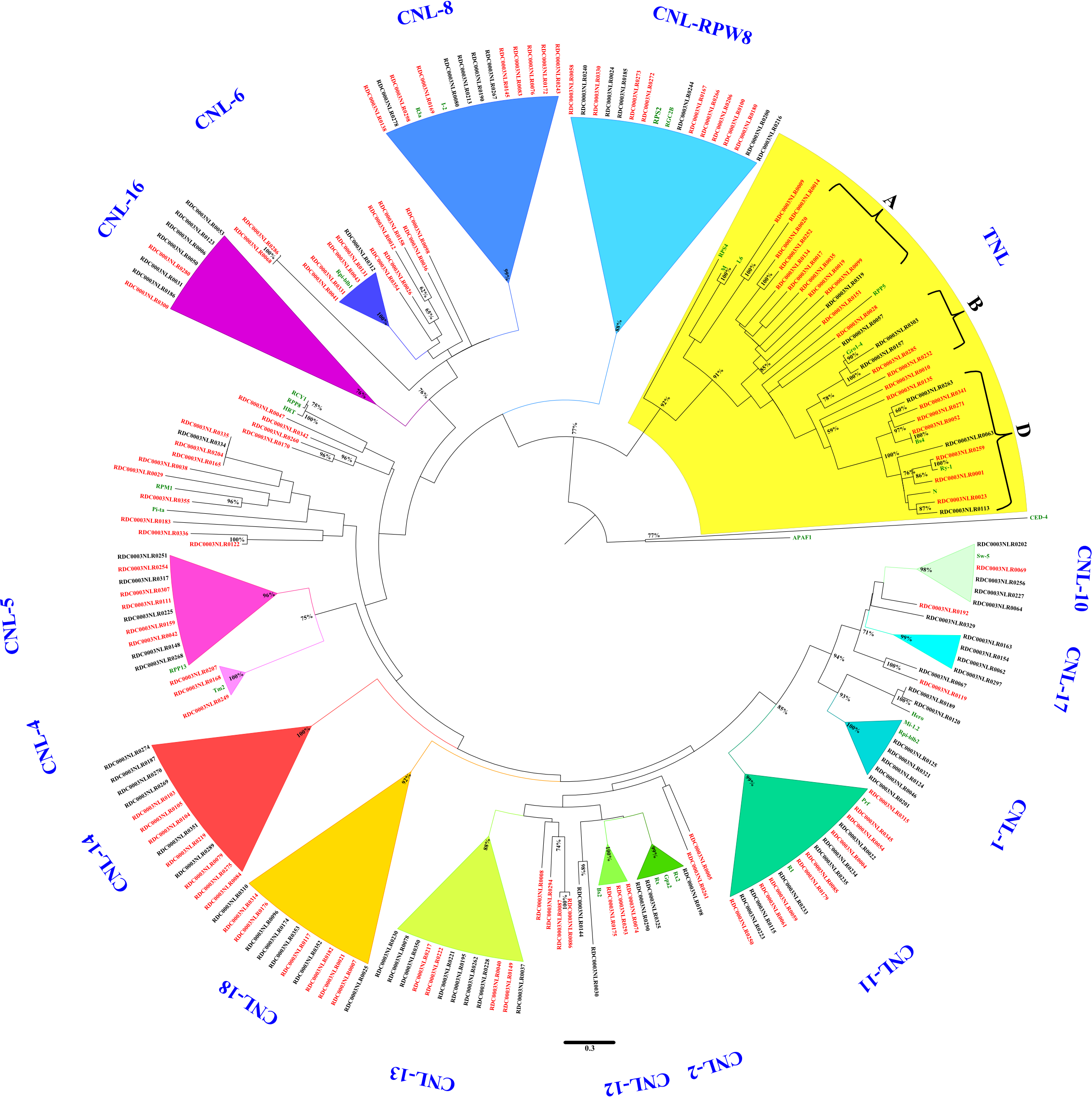

Supplement: Additional file 6 — Phylogenetic tree of Solanum pimpinellifolium LA1589 NB-ARC domains. Evolutionary analyses were performed like in Heinz 1706, on the basis of the NB-ARC domain of 222 rennotated NB-LRRs. Labels show the gene IDs red for expressed NB-LRR genes; black for not-expressed genes. [file 1471-2229-14-120-S6.png]
